# Case Report: Altered with PRES

**DOI:** 10.21980/J8NW73

**Published:** 2021-04-19

**Authors:** Fatima Dema, David C Feldman

**Affiliations:** * Morristown Medical Center, Department of Emergency Medicine, Morristown, NJ

## Abstract

In this case, we discuss a 51-year-old female with history of anxiety, depression, and alcohol abuse who presented for altered mental status, focal neurological deficits, and seizure. She was found to be significantly hypertensive. Non-contrast computed tomography (CT) scan imaging of the head revealed changes suggestive of posterior reversible encephalopathy syndrome (PRES), which was ultimately confirmed by MRI imaging. The patient was treated appropriately with anti-hypertensive therapy and seizure prophylaxis. Due to the prompt recognition of PRES and immediate management, the patient was able to make a favorable neurological recovery. This case highlights the importance of including PRES as part of a wide differential diagnosis for a patient with altered mental status, significantly elevated blood pressure, and focal neurological deficits. Early recognition and treatment of PRES can improve neurological outcomes and quality of life for patients.

**Topics:**

Altered mental status, seizure, hypertensive emergency, posterior reversible encephalopathy syndrome, PRES.[Fig f1-jetem-6-2-v5]

**Figure f1-jetem-6-2-v5:**
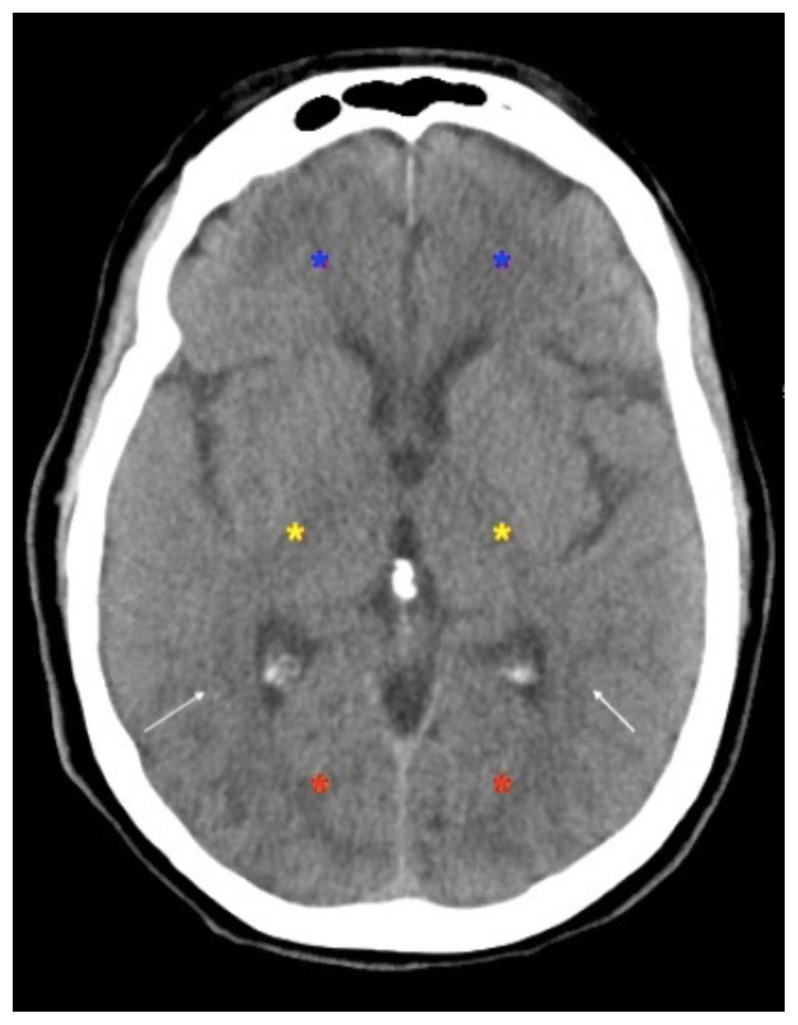
Video Link: https://youtu.be/7_52fos388E

## Brief introduction

Posterior reversible encephalopathy syndrome (PRES) is a life-threatening neurological disorder comprised of multiple neurological symptoms as well as characteristic neuroimaging findings of posterior cerebral white matter changes. The exact pathogenesis and epidemiology of PRES is still unknown due to its low incidence rate, although its prompt diagnosis and treatment is important in order to prevent permanent neurological damage.^1^ In this case report, we describe a patient with altered mental status and focal neurologic findings who was found to have PRES.

## Presenting concerns and clinical findings

A 51-year-old female with a past medical history of anxiety, depression, and alcohol abuse presented from home for altered mental status. According to medics, the patient was found unresponsive at home by her daughter. When medics arrived on the scene, the patient was noted to have left-sided paralysis with leftward gaze preference and found to be hypertensive. En route to the hospital, the patient was noted to have a witnessed generalized tonic-clonic seizure, which resolved after administration of Versed. Upon arrival to the emergency department, the patient was noted to be confused, dysarthric, with left-sided paralysis and leftward gaze preference. Further information obtained by the daughter revealed the patient had nausea, vomiting, and diarrhea a few days prior to presentation but otherwise was feeling generally well.

## Significant findings

On arrival, the patient was noted to have significant weakness on the left side. Motor strength exam revealed 2 out of 5 strength in the left upper and lower extremities and 4 out of 5 strength in the right upper and lower extremities. She continued to have a leftward gaze preference with dysarthria. Neuro exam was otherwise limited secondary to the patient intermittently not following commands and being agitated.

On vitals, the patient was found to be consistently hypertensive to the 230s/160s. Point-of-care glucose was within normal limits. Noncontrast CT imaging of the head revealed no acute intracranial hemorrhage or evidence of ischemic stroke, but was remarkable for areas of biparietal subcortical lowattenuation (white arrows), concerning for PRES. Patient subsequently underwent CT angiogram imaging of the head with perfusion which revealed no large vessel occlusion.

## Patient course

The patient’s clinical history and concerning CT image findings prompted urgent treatment for possible PRES syndrome. For the hypertension, the patient was treated with Labetalol 10mg intravenously (IV) and subsequently started on Nicardipine IV drip. For seizure prophylaxis, the patient was loaded with Keppra 1g IV. Neurosurgery was emergently consulted. As per their recommendations, the patient was transferred to a neurosurgical intensive care unit. She had repeat CT head imaging at the transfer facility which showed progressive subcortical edema in the bilateral parietal lobes (white arrows), as well as in the bilateral frontal (blue asterisks), posterior temporal (yellow asterisks), occipital lobes (red asterisks), and cerebellum. The patient had a follow up MRI brain with and without contrast, which revealed extensive subcortical edema present throughout both cerebral and cerebellar hemispheres, most prominent along the bilateral posterior temporal and occipital lobes, compatible with PRES. There was no restricted diffusion to suggest acute infarction. Patient also had a lumbar puncture performed, which was unremarkable. She was also evaluated by the Epilepsy service and placed on video EEG monitoring. She had no noted seizure activity and, therefore, was not placed on any anti-epileptic medications.

For the significant hypertension, the patient was transitioned from Nicardipine IV drip to a combination of oral agents, including amlodipine, captopril, lisinopril, hydralazine, and metoprolol. Her intensive care unit (ICU) stay was complicated by a right lower lobe pneumonia. She was treated with IV antibiotics for a presumed aspiration pneumonia secondary to the pre-hospital seizure.

While in the intensive care unit, the patient had significant improvement in her neurological exam. Her speech became fluent with complete resolution of the dysarthria. All visual fields were intact with no gaze preference appreciated. On motor strength examination, the patient regained strength to 5 out 5 in all four extremities with stable, steady gait. She was evaluated by physical and occupational therapy and deemed safe for discharge with home physical therapy.

## Discussion

In this case report, we discussed the clinical presentation, diagnosis, management, and prognosis of a patient with PRES. Historically, the syndrome dates back to 1996, when the first case series was reported by Hinchey, et al. The series consisted of 15 patients with a mixture of neurological complaints, such as headache, altered mental status, and seizure with neuroimaging findings consist with bilateral posterior cerebral and cerebellar edema. Interestingly, the symptoms were noted to resolve after about two weeks, thereby classifying these patients’ condition as reversible. Common inciting factors noted were uncontrolled hypertension and immunosuppressive therapy.^2^

The pathogenesis of PRES still remains unclear. There are two main mechanisms that have been proposed: endothelial dysfunction and hypertension with autoregulatory failure. Endothelial dysfunction theory has commonly been implicated in the case of cytotoxic agents, such as immunosuppressive medications or chemotherapeutics, sepsis, and preeclampsia.^3^ The second theory postulates that excessive hypertension causes loss of cerebral autoregulation resulting in arteriolar dilation and cerebral hyperperfusion. This increased blood flow leads to destruction of the blood-brain barrier, causing fluid extravasation and therefore edema of the brain parenchyma. Typically, PRES patients have significantly elevated blood pressures compared to those with presumed endothelial dysfunction secondary to presumed auto regulatory failure.^4^

The diagnosis of PRES requires brain imaging. The initial imaging of choice is typically CT head without contrast due to its quick accessibility. Although CT imaging can depict characteristic neuroimaging abnormalities of PRES, they are better depicted on MRI imaging, making MRI an essential component of a full workup of this syndrome. Typical neuroimaging findings consist of bilateral areas of cerebral and cerebellar edema, particularly in the parietal and occipital regions, as was depicted by our patient. The improvement and/or resolution of neuroimaging findings has been shown to correlate with the improvement in clinical symptoms over time.^5^,^6^ Lumbar puncture may be considered as part of the work up in order to rule out other neurological etiologies, such as meningitis, although it is not required for the diagnosis of PRES.^7^

The treatment of PRES can be generalized to management of hypertension and seizures. Blood pressure is controlled as per hypertensive emergency protocol, with initial agents such as intravenous Labetalol and Nicardipine, followed by transition to oral agents for long-term control. Seizure prophylaxis is accomplished through intravenous Levetiracetam or Lacosamide. Further treatment is tailored according to the underlying cause, such as discontinuation of any cytotoxic or immunosuppressive agent or delivery of the baby in preeclampsia.^8^

Although death and permanent neurological disability have been reported from PRES, most published case reports describe a favorable prognosis for patients suffering from this syndrome. Clinical symptoms, as well as radiological signs, usually improve and/or resolve anywhere from days to weeks after complete treatment, as was shown by our patient.^9^

In summary, this case report highlights the importance of emergency medicine providers to be familiar with the presentation, diagnosis, and initial treatment of PRES. Despite its rarity, timeliness of intervention and management is critical for improved long-term neurological outcomes.

## Supplementary Information




