# Supplementary figures and images for: Case Report: Altered with PRES
Source: J Educ Teach Emerg Med. 2021 Apr 19;6(2):V5–7. doi: 10.21980/J8NW73 (PMC10332781; doi:10.21980/J8NW73)

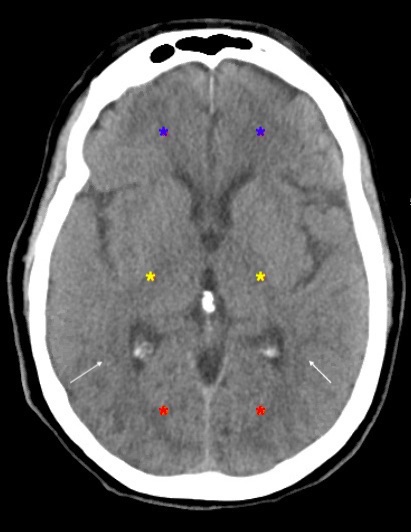

Supplement: Supplementary file 1 [file jetem-6-2-v5-supp1.jpeg]
